# Detecting Changes of a Distant Gas Source with an Array of MOX Gas Sensors

**DOI:** 10.3390/s121216404

**Published:** 2012-11-27

**Authors:** Sepideh Pashami, Achim J. Lilienthal, Marco Trincavelli

**Affiliations:** Centre for Applied Autonomous Sensor Systems,Örebro University,Örebro SE-70182, Sweden; E-Mails: achim.lilienthal@oru.se (A.J.L.); marco.trincavelli@oru.se (M.T.)

**Keywords:** MOX sensor, open sampling system, sensor selection, change point detection

## Abstract

We address the problem of detecting changes in the activity of a distant gas source from the response of an array of metal oxide (MOX) gas sensors deployed in an open sampling system. The main challenge is the turbulent nature of gas dispersion and the response dynamics of the sensors. We propose a change point detection approach and evaluate it on individual gas sensors in an experimental setup where a gas source changes in intensity, compound, or mixture ratio. We also introduce an efficient sensor selection algorithm and evaluate the change point detection approach with the selected sensor array subsets.

## 1. Introduction

Change point detection algorithms that analyze the response of an array of gas sensors and detect a change in the exposure of the array to a gas mixture can bring a significant leap forward in the construction of systems for monitoring of hazardous or pollutant gaseous compounds. Up to now, most of the work with gas sensors in an open sampling system (OSS) (*i.e.*, without a sensing chamber that controls the exposure of the sensors to the gas and other variables like temperature and humidity) has been developed under simplified assumptions such as steady air flow and a gas source emitting a single compound with constant emission rate for the whole duration of the experiments. Unfortunately, these assumptions rarely hold in scenarios of interest for practical applications, like monitoring of industrial production plants [1], landfills [2] and demining [3]. In such applications, gas sensors are preferably deployed in an open sampling system since a quick and continuous response is often crucial, and restrictions in costs and payload pose stringent limitations on the hardware that can be considered. Moreover, it is often desirable to expose sensors directly to the environment to be analyzed since the dynamic response of the gas sensors contains crucial information on the gas plume and in particular on the location of the gas source [4]. These aspects acquire additional relevance when the gas sensors are mounted on mobile robots that have to perform tasks like gas distribution mapping or gas source localization [5,6]. However, the OSS configuration entails additional problems [7,8]. The lack of control over the environmental conditions results in a low reproducibility of experiments with gas sensors in OSS configuration. This is mainly due to unpredictable fluctuations in the concentration profile that are due to the mechanisms of gas dispersion, which in natural environments—characterized by a high Reynolds number—are dominated by turbulence and advection [9]. A second key problem is that the dynamics of most gas sensing technologies are slow compared with the fast fluctuations in the concentration profile to which the sensor are exposed.

The problem we address in this work is the detection of changes in the activity of a distant gas source from the response of an array of metal oxide (MOX) gas sensors deployed in an open sampling system. In an attempt to move towards realistic scenarios, we consider a single gas source that changes compound, intensity or mixture of compounds in the course of a single experiment. MOX sensors are the most common gas sensors used in OSS [10], mainly because of their commercial availability and the high sensitivity to non-hazardous compounds like alcohols (which facilitate experiments). However, MOX gas sensors suffer from long response and recovery times and, consequently, the response seldom reaches a steady state when used in an OSS. The change detection problem addressed in this work is thus particularly hard since true changes have to be distinguished from mere fluctuations in the sensor response due to turbulent gas dispersion.

The presented algorithm for change detection is derived from the well known Generalized Likelihood Ratio algorithm [11] and it is evaluated using three performance measures, namely the detection rate, the false alarm rate, and the delay of detection. First, the performance of the algorithm considering a single gas sensor is analyzed, then the scope is extended by applying the algorithm to multiple sensors. An efficient approach to select a subset of the available sensors in order to maximize the change point performance is proposed. The sensor selection policy is based on a trade-off, which can be controlled through a parameter, between the accuracy and the speed of detection.

The rest of this paper is organized as follows. Section 2 presents related works both for what concerns change point detection in a multivariate time series and gas sensing with an OSS. Section 3 describes the experimental setup with which the algorithms have been tested. Section 4 details the change point detection algorithm. Section 5 describes the sensor selection policy. Results are then presented in Section 6, first for what concerns the single sensor change point detection and then considering the change point detection using multiple sensors. Finally, Section 7 draws the conclusions and gives an outline of future works.

## 2. Related Works

The detection of changes in the activity of a gas source based on the response of an array of MOX gas sensors has, to the best of our knowledge, not been studied so far. However, we can relate this work to the study of change point detection in the domain of the analysis of multivariate time series. Indeed the response of an array of gas sensors sampled at constant intervals can be considered as a multivariate time series. Change detection in multivariate time series has a wide range of applications such as quality control, segmentation of signals, monitoring of production processes or vehicles. It is obvious that such a wide range of applications corresponds to the development of very different solutions for the change point detection problem. Variations span from on-line to off-line algorithms, multivariate or univariate and detecting additive or multiplicative changes [11].

Probably the simplest solution for change detection is detecting when the measurements fall out of a predefined range. This solution has been proposed for quality control applications [12]. Other techniques estimate change points by investigating the behaviour of the measurements of the time series before and after a hypothetical change point. These techniques often use statistical approaches both in a model based and model free fashion. The most common algorithms, inspired by frequentist statistics, are the Generalized likelihood Ratio (GLR) test [11], the Marginalized Likelihood Ratio (MLR) [13] and the CUmulative SUM (CUSUM) algorithm [11]. If a prior on the time of the change point can be assumed, Bayesian inspired algorithms have also been proposed [14]. Finally, change point detection algorithms inspired by machine learning approaches such as one class Support Vector Machine [15] have been proposed as well.

In this paper we consider change detection at a single location (even in the case where we consider multiple sensors we assume that they are exposed to the same concentration due to spatial proximity). Actual applications will either combine the gas sensor with some means of mobility (for example by mounting the sensors on a mobile robot) or consider a sensor network. The only work proposed in this field, to the knowledge of the authors, is dealing with a sensor network detecting anomalies in the gas distribution in coal mines [16]. A Bayesian Network is proposed to trigger alarms in case the sensor response at different nodes is anomalous, However, the approach in [16] is mainly concerned with the spatial distribution of the gas concentration and neglects many problems entailed with chemical sensing, including the cross correlation among the response of gas sensors of different type.

## 3. The Experimental Setup

**Figure 1.**
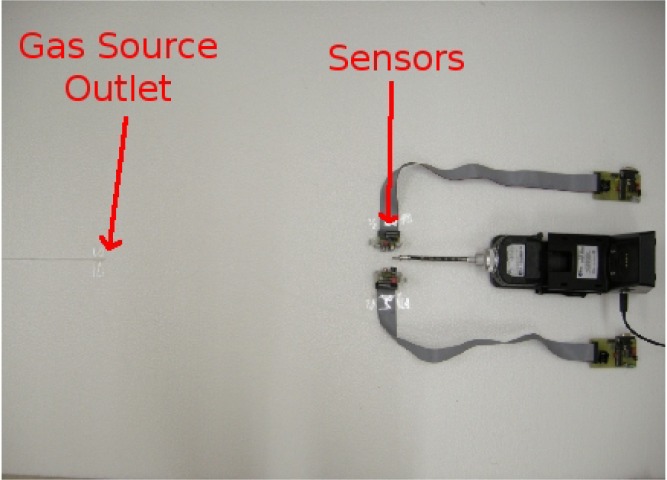
Picture of the gas source and the sensor array. The device in the middle of the picture is a Photo Ionization Detector (PID), which has not been considered in this work.

We carried out experiments in a 5 × 5 × 2 *m*^3^ closed room with static sensors where an artificial airflow of approximately 0.05 *m/s* is induced. The airflow is created using two arrays of four fans (standard microprocessor cooling fans), one placed on the floor and one on the wall. The gas source is an odour blender, a device developed by Nakamoto *et al.*[17], which allows fast switches in between different mixtures of compounds with a variable concentration. The outlet of the olfactory blender is placed on the floor 0.5 *m* upwind with respect to an array of 11 commercial metal oxide gas sensors from Figaro Engineering [18] and e2v Technologies [19]. Table 1 presents a list of the gas sensors models together with the nominal target compounds as declared by the producer on the data sheet of each sensor. The selected sensors have overlapping sensitivity and they respond to a wide range of target compounds. The airflow at the outlet of the odour blender is set to 1 L/min. The sensors are sampled at 4 Hz. Figure 1 shows a picture of the experimental setup.

The two compounds selected for these experiments are ethanol and 2-propanol. Both ethanol (molecular weight 46 g/mol) and 2-propanol (molecular weight 60 g/mol) are heavier than air (average molecular weight 29 g/mol), and therefore will tend to create a plume at the ground level.

The two substances have a similar saturated vapor pressure, namely 5.8 kPa for ethanol and 4.2 kPa for 2-propanol, which means that they have a similar tendency to evaporate. Moreover, MOX gas sensors have comparable sensitivity to the two substances. This is important in order to obtain similar sensor responses for both analytes, thus avoiding to address a trivial instance of the change detection problem.

**Figure 2.**
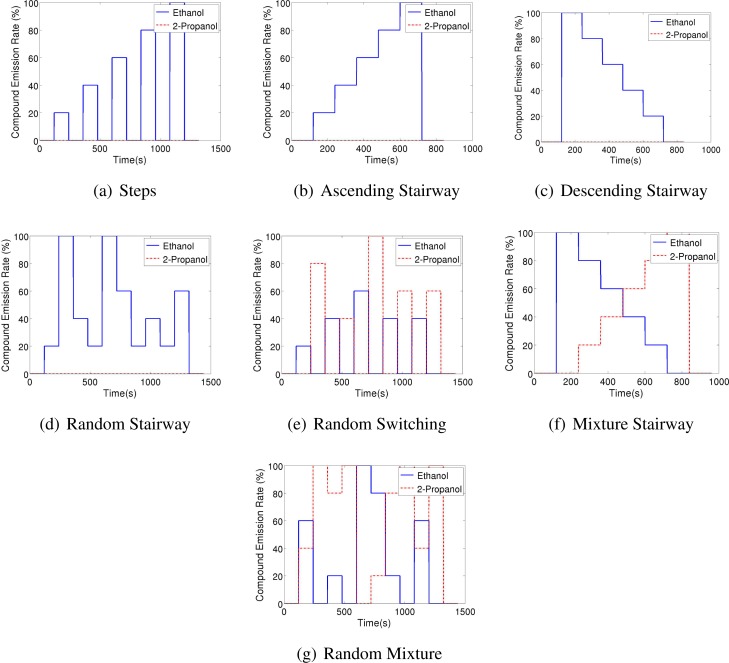
Gas source emission strategies. Strategies (**a–d**) are displayed only for ethanol (they are repeated identically also with 2-propanol as target gas). For the randomized strategies, *i.e.*, (**d**), (**e**), and (**g**), one exemplary instance is displayed.

In order to create a database that allows to study the dynamic behaviour of the sensors when consecutively exposed to different analytes, seven different odour emitting profiles have been applied. For all these profiles the gas source emits clean air for two minutes and the signal of sensors during this period is assumed as a baseline. Also, at the end of all the experiments the source emits clean air for 2 minutes. Figure 2 shows the intensity profile for the gas source in the various emission strategies. A total of 54 experimental runs have been performed.

The control signal of the odour blender is used as ground truth for the change point time and provides the time at which the source changes the emission modality. However, in order to know the change point time at the sensors’ location, we need to estimate the time it takes the gas to travel from the gas source to the sensor location. Since the sensors are placed 0.5 *m* away from the location of the source outlet and a steady air flow of 0.05 *m/s* is induced, the delay time between change times at source and sensor location is estimated to be 10 *s*.

## 4. Change Point Detection Algorithm

**Figure 3.**
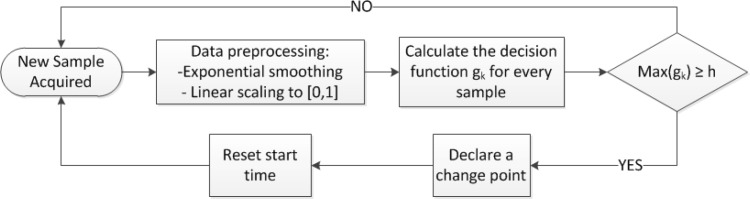
Block diagram explaining the on-line GLR algorithm.

No prior information is assumed about the position of the change points. We further assume that no information about the length of the monitoring process is available and have therefore chosen an algorithm that processes data on-line. Because of these reasons, we are using an adaptation of the well-known Generalized Likelihood Ratio (GLR) algorithm [11]. The presented algorithm is schematically shown in Figure 3 and described in the following sub-sections: data preprocessing, GLR algorithm and performance measures used for evaluation.

**Figure 4.**
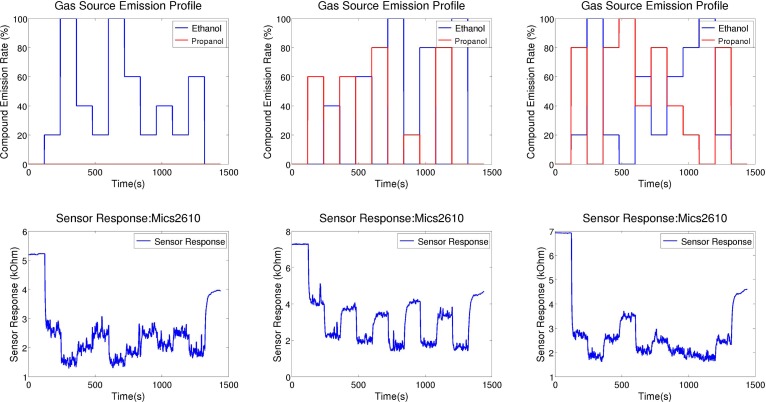
Three experimental runs that show changes in gas concentration (**left**), compound (**middle**), and mixture ratio (**right**) at gas source. The three top figures show the emission profile of the gas source, while the three figures at the bottom show the response of sensor MiCS2610.

The changes we consider in this work are due to a change in the intensity of the gas source, a change in the chemical compound the gas sensor is exposed to, or a change in the gas mixture. These different types of changes are visualized and shown together with the corresponding sensor response in Figure 4. Notice the fluctuations in the sensor response, which are due to turbulent dispersion and not due to changes in the emission modality of the gas source. This is a key reason for which change point detection is non-trivial.

### 4.1. Data Preprocessing

Before running the GLR algorithm to detect change points, the raw sensor measurements are preprocessed using a low pass filter and a normalization operation described in the two following paragraphs.

#### 4.1.1. Exponential Smoothing (Low Pass Filter)

The gas sensor response contains noise due to the electronics of the acquisition system. To dampen this noise, the sensor response is filtered using an Exponential Moving Average (EMA) filter [20]. The EMA filter is an infinite impulse response filter that applies weighting factors that decrease exponentially. For a time series *x*_0_, …, *x_N_* the EMA response can be calculated recursively using the following equations:

(1) $$s_{0}=x_{0},$$

(2) $$s_{t}=\alpha s_{t-1}+(1-\alpha )x_{t},\;\;\;\;\;\;\;\;\;\forall t>0$$

where *x*_0_, *x_t_* is the raw sensor signal, *s*_0*...N*_ is the smoothed sequence and *α* is a smoothing factor. The factor *α* is always between 0 and 1. Values of *α* close to 0 result in an aggressive smoothing while values of *α* close to 1 nearly preserve the original time-series. We select a value of *α* = 0.9 in our experiments since this value gives a cut-off frequency for the filter of 0.44 *Hz*. Since this cut-off frequency is higher than the one applied by the MOX sensors themselves, the EMA filter mainly removes electronic noise without slowing down the actual response of the sensors.

#### 4.1.2. Normalization

Due to differences in the sensing surface, different models of MOX sensors exhibit a different dynamic range. This means that some of the sensors, when responding, change their resistance value only a few Ohms while others vary hundreds or even thousands of Ohms. Thus, before running change point detection algorithms, we make the dynamic ranges of the sensors comparable by normalizing the response *s*_1*...N*_ of each sensor to the interval [0, 1] using the following linear transformation:

(3) $${s^{\prime }}_{t}=\frac{s_{t}-{\text{min}}_{1...N}s_{i}}{{\text{max}}_{1...N}s_{i}-{\text{min}}_{1...N}s_{i}}$$

where *ś*_1...*N*_ is the normalized sensor response.

### 4.2. GLR Algorithm

Given the smoothed and normalized sensor response *ś*_1...*k*_ where *k* is the current time index, the GLR algorithm calculates the likelihood ratio between the hypotheses of having a change point at sample *j* versus the hypothesis of not having a change point:

(4) $$\Lambda_{j}^{k}=\frac{\prod_{i=1}^{j-1}\;p_{\theta_{0}}({s^{\prime }}_{i})\;\prod_{i=j}^{k}\;p_{\theta_{1}}\;({s^{\prime }}_{i})}{\prod_{i=1}^{k}\;p_{\theta_{0}}\;({s^{\prime }}_{i})}=\prod_{i=j}^{k}\frac{p_{\theta_{1}}\;({s^{\prime }}_{i})}{p_{\theta_{0}}\;({s^{\prime }}_{i})}.$$

The likelihoods are based on a parametric probability distribution function *p_θ_*, which is governed by a set of parameters *θ*. Since no prior information on the sensor noise is available, the most natural choice for *p_θ_* is the Gaussian distribution, which is governed by two parameters, namely the mean and the variance. *θ*_0_ denotes the mean/variance estimated using all samples in the time interval to be checked for change points. *θ*_1_ denotes the mean/variance estimated using only the samples collected after a hypothetical change point *j*. In this work we are interested in detecting level shifts in the response of one or more gas sensors and therefore the variance is assumed to be constant and is estimated considering all the samples in the considered interval.

For numerical reasons, it is more convenient to calculate the log-likelihood value $S_{j}^{k}$ instead of the likelihood $\Lambda_{j}^{k}$ itself:

(5) $$S_{j}^{k}=\sum_{i=j}^{k}\text{ln}\frac{p_{\theta_{1}}\;({s^{\prime }}_{i})}{p_{\theta_{0}}\;({s^{\prime }}_{i})}.$$

The decision function *g_k_* is obtained by taking the maximum with respect to possible change point times *j*:

(6) $$g_{k}=\underset{i\leq j\leq k}{\text{max}}\;S_{j}^{k}$$

If *g_k_* is above a pre-selected threshold *h*, then a change point is declared and the data collected before the change point are not considered any longer to detect new change points. In case a change point is detected, *k* is the *alarm time* and *ĵ,* which is the value of *j* for which $S_{j}^{k}$ attained its maximum, corresponds to the *detected time of change*. Otherwise, a new sample is acquired, the indexes are updated, and the change point detection process is repeated. Notice that the algorithm is readily extended to a multivariate setting by considering multivariate instead of univariate Gaussian distributions.

### 4.3. Performance Measures

**Figure 5.**
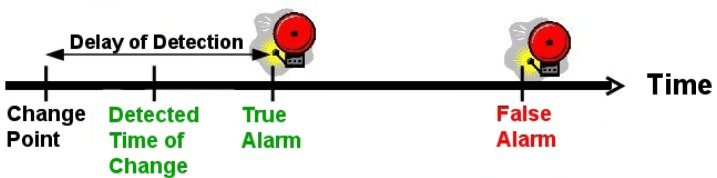
Example in which one true alarm and one false alarm are triggered.

To evaluate the change detection algorithm, three performance measures were used: the true alarm ratio, the false alarm ratio and the delay of detection. Before providing a definition of the performance measures, we define the concepts of true alarm, false alarm, and delay of detection. A *true alarm* is defined as the first alarm after a change point. Any other alarm coming after the true alarm and before the next change point is defined as a *false alarm*. The *delay of detection* is defined as the difference between the alarm time of a true alarm and the time of the change point. Figure 5 shows a graphical representation of these concepts.

The first performance measure we consider is the true alarm ratio (TAR), which is given by the total number of true alarms divided by the number of change points. Clearly, the value of this performance measure is bounded between 0 and 1. The second performance measure is the false alarm ratio (FAR), which is calculated as the total number of false alarms divided by the number of change points. Notice that this performance measure is unbounded. The third performance measure is the mean delay of detection (MDD) and is defined as the average of the delay of detection.

## 5. Sensor Selection

**Figure 6.**
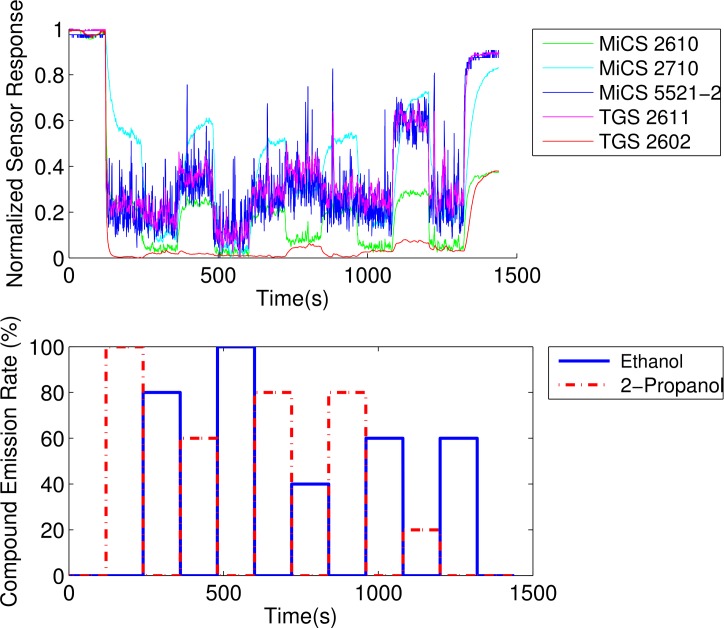
Normalized Response of five MOX sensors to a series of compound switches.

MOX gas sensors are characterized by high correlation in their response [21]. This entails that the information provided by an array of MOX sensors is highly redundant. As an example, Figure 6 shows the response of five of the sensors in the array to a series of compound switches. This plot evidently shows the high correlation among the responses of the sensors.

In order to reduce power consumption and the risk of suffering from the *curse of dimensionality*, it is necessary to select the minimum number of sensors that gives optimal performance. Among various ways proposed in literature for selecting an optimal subset of features, or sensors in our case [22,23], the Quadratic Programming Feature Selection (QPFS) method suggested by Lujan *et al.* in [24] is theoretically well founded and computationally very efficient, since it is based on convex optimization. The QPFS method attempts to solve the following optimization problem

(7) $$\underset{x}{\text{minimize}}\;\;\;\;\;\;\;\;\;\;(1-\alpha )x^{\prime }Qx-\alpha F^{\prime }x$$

(8) $$\begin{array}{ll} \text{subject}\;\text{to} & x≽0\\ & e^{\prime }x=1 \end{array}$$

If we consider a problem with *M* sensors, **x** is a vector of length *M* that represents the importance of each sensor for the problem at hand, **Q** is an *M* × *M* matrix that measures the redundancy of each pair of sensors, **F** is a vector of length *M* that measures the relevance of each sensor for the task at hand, and **e**′ is a vector of ones of length *M*. QPFS ranks the sensors trading off maximum relevance and minimum redundancy and the parameter *α* ∈ [0, 1] acts as a regulator of this trade-off.

In this work, the matrix **Q** is formulated calculating the Pearson’s coefficient between each pair of sensors in the Steps experiments. Therefore redundancy is in this case expressed by the linear correlation among the sensor responses. The vector **F** has to express the relevance of each sensor which, according to our performance measures, is a trade-off between the ease in detecting change points and the speed of detection. As a measure for the ease of detecting change point, we use the average Fisher Index of the sensor response considering as classes the sensor response to different gas source emission rates. The Fisher Index is an index of separation between classes that is calculated as the distance between the mean value of the samples belonging to each class divided by the mean standard deviation of the samples in the class. As a measure of the speed of each sensor, we use the average of the inverse time constant estimated in the decay phases of the Step experiments. We decided to use the time constants of the decay phase since they are much larger than the ones of the rise phase. Therefore, the vector **F** can be expressed by the following equation:

(9) $$F=(1-\beta )\frac{F_{\mathrm{FI}}}{e^{\prime }F_{\mathrm{FI}}}+\beta \frac{F_{\mathrm{ITC}}}{e^{\prime }F_{\mathrm{ITC}}}$$

where **F_FI_** is a vector containing the Fisher Index for each sensor, **F_ITC_** is a vector containing the inverse time constant for each sensor, and *β* is a parameter for controlling the trade-off between the speed of the sensor and the change of the sensor response in correspondence of a change point.

## 6. Results

**Figure 7.**
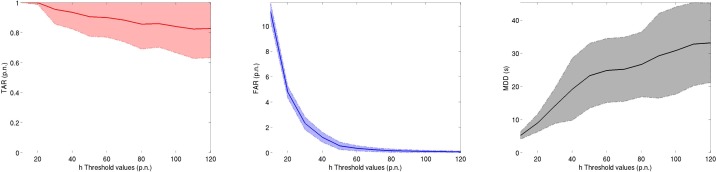
Evolution of the performance measures for a varying threshold *h*. Lines are the mean value of the performance measure across the 55 different experiments, while shaded areas represent plus/minus one standard deviation.

Figure 7 shows the performance measures of the proposed algorithm when varying the threshold value *h*. As expected, the selection of the threshold value governs a trade-off between the change point detection ratio (TAR, prioritized by low threshold values) and the false alarm ratio (FAR, prioritized by high threshold values). Also, the delay of detection (MDD) generally increases with the threshold. This is within expectation since higher thresholds require the collection of more evidence in the data in order to trigger an alarm. Table 2 reports the time constants and Fisher Indices for each of the sensors. The time constants have been calculated by fitting an exponential to each of the transients induced in the Steps experiments. Since no significant variation in the time constant was observed regarding the concentration that the sensors were exposed to, the mean of all the experiments can be considered as a reliable estimator of the time constant of the sensors. The time constants in Table 2 were used in the sensor selection algorithm as an indicator of the speed of a sensor.

Section 6.1 presents the results obtained with the proposed change point detection algorithm when considering a single sensor at the time. Section 6.2 presents the results of the proposed sensor selection strategy and Section 6.3 concludes with the results obtained with those sensor combinations that were selected by the sensor selection strategy.

### 6.1. Single Sensor Performance

**Figure 8.**
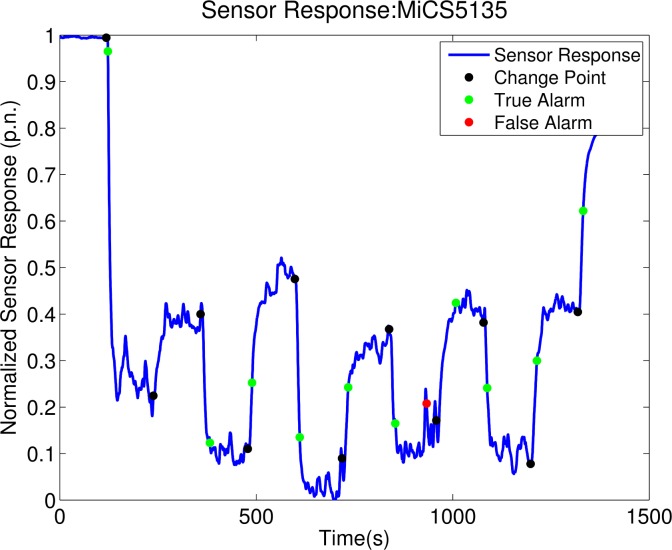
Result of the execution of the proposed algorithm for an experiment where the gas source was changing the emitted compound. The sensor is the MiCS 5135 and the threshold *h* is set to 77 (as in Table 3). In this case, one of the change points was not detected and one false alarm was triggered.

Table 3 reports the results of the single sensor change point detection when the false alarm ratio (FAR) is set to 0.1. Sensor MiCS 5135 is the one that attains the highest overall TAR with also the shortest delay. Figure 8 shows an example of the execution of the proposed algorithm on the response of the sensor MiCS 5135. All the other sensors have a comparable overall MDD. Sensor TGS 2602 is the one that attains the worst overall TAR and it was found to be particularly bad in detecting changes in the mixture of the two compounds.

The sensors MiCS 5135 and MiCS 2610 prove to be particularly good in detecting changes in compound corresponding to the highest TAR values. In addition, the sensor MiCS 2610 has also the lowest MDD for what concerns changes in compound. Together with the sensor MiCS 5135, the sensors MiCS 2610 and MiCS 2710 are the only two other sensors that obtain a TAR higher than 0.7 for changes in mixture. Concerning changes in concentration, only the sensors MiCS 5135 and MiCS 5121 were found to be both efficient and fast (high TAR and a low MDD).

### 6.2. Sensor Selection Results

**Figure 9.**
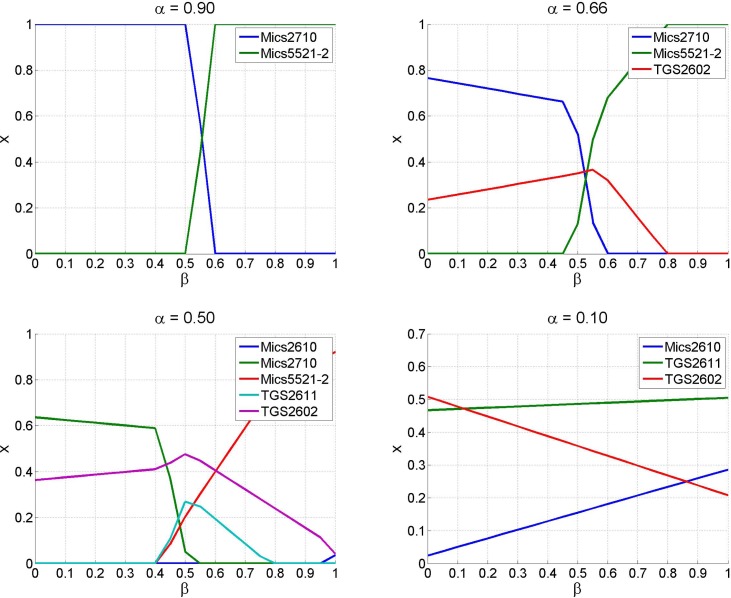
Results of the sensor selection algorithm for various settings of *α* and *β*. Each sub-figure corresponds to a different value of *α*, where large values of *α* favour subsets of relevant sensors while small values of *α* favour subsets of uncorrelated sensors. Low values of *β* favour sensors that detect change points easily, while high values of *β* favour quick sensors.

The proposed sensor selection method has two parameters that need to be selected. The parameter governs the trade-off between relevance and redundancy of the selected sensor set, while *β* defines the relevance of a sensor as a trade-off between speed and ease of detecting change points. Figure 9 shows the importance of sensors for some configurations of *α* and *β*. It is worth noticing how for high values of only the most relevant sensor is selected, which is the quickest sensor when *β* is high, or the most discriminative sensor when beta is low. On the other hand, when *α* is small, and therefore sets of uncorrelated sensors are preferred, the value of *β* that governs the trade-off of the relevance term is much less influential.

Table 4 lists the different sensor subsets selected for different values of the parameters *α* and *β*. Notice that the configurations containing a single sensor, *i.e.*, those for which *α* = 0.9 and *β* ∈ [0, 0.5] ∪ [0.6, 1], have been omitted since they fall back into the single sensor case. It is also worth noticing how all the sensor subsets contain a very low number of sensors, maximum three, compared with the eleven sensors present in the array. Likely, this is due to the high correlation among the responses of MOX gas sensors. Using more than two or three sensors does only increase redundancy of the array without increasing relevance.

### 6.3. Results for the Selected Sensor Subsets

Table 5 reports the performance obtained for the selected subsets of the array when the false alarm ratio (FAR) is set to 0.1. First it comes to notice that the selected thresholds *h* are in general higher than in the single sensor case. Also the selected thresholds *h* are higher for sets of three sensors compared with sets of two sensors. This can be due to the fact that larger arrays imply the estimation of higher dimensional Gaussian distributions, which are more prone to overfitting than lower dimensional ones. In order to avoid overfitting, higher thresholds tend to be selected, which imply the need of more evidence for declaring a change point.

All the considered set of sensors are outperforming the single sensors in the overall performance evaluation, since they manage to achieve a TAR that is comparable to the one of the best single sensors while having a better MDD. It is particularly interesting to notice how the combinations that do not include the MiCS 2710 sensor, which was one of the sensors achieving the highest overall TAR but at the same time suffering from a high MDD, manage to achieve a high TAR *and* a low MDD.

It is further worth noticing how the difference in performance for the selected sets of sensors is much smaller than the difference among the performance of single sensors. All the sensor sets seem to be able to perform uniformly well in all the tasks.

## 7. Conclusion

In this paper we address the problem of detecting changes in the activity of a distant gas source from the response of an array of metal oxide (MOX) gas sensors deployed in an open sampling system. First, we describe an approach for change point detection that is inspired by the well known Generalized Likelihood Ratio (GLR) algorithm. The algorithm can be applied using either the output of a single sensor or the output of a set of sensors. We carried out a substantial number of tests, based on which we evaluate the performance of the proposed algorithm in both cases. Results obtained with a single sensor are presented in Section 6.1 while the results obtained with several sensors are presented in Section 6.3. The sensor subsets were selected using a novel and efficient sensor selection strategy that is presented in Section 5 and evaluated in Section 6.2.

Our results show that, given a fixed rate of false alarms (set to be 0.1 in our case), the selected sets of sensors obtain a detection rate comparable to the best single sensors, however with a significantly lower delay of detection. In particular, the subsets including a fast sensor like the MiCS 5521 have demonstrated short delays of detection. It is noteworthy that these configurations were obtained when quick sensors were included in the array by the proposed sensor selection method, which trades off relevance, ease of change point detection and the quickness of a sensor.

Future work will include the development of methods for automatic selection of the threshold *h* and testing of the proposed algorithms on more general setups, for example when the sensor array is mounted on a mobile robot or when it is deployed in an outdoor uncontrolled environment. An example of a scenario where it can be very interesting to test the change point algorithms in the future is the Air Quality Egg project for monitoring pollution in towns (http://www.kickstarter.com/projects/edborden/air-quality-egg).

## Figures and Tables

**Table 1.** Metal Oxide gas sensors used in the various experimental setups.

**Gas Sensor Model**	**Indoor Static Sensors**	**Target Gases**

Figaro TGS 2600	2	Hydrogen, Carbon Monoxide
Figaro TGS 2602	1	Ammonia, Hydrogen Sulphide, VOCs
Figaro TGS 2611	1	Methane
Figaro TGS 2620	1	Carbon Monoxide and Organic Solvents
e2V MiCS 2610	1	Ozone
e2V MiCS 2710	1	Nitrogen Dioxide
e2V MiCS 5521	2	Carbon Monoxide, Hydrocarbons, VOCs
e2V MiCS 5121	1	Carbon Monoxide, Hydrocarbons, VOCs
e2V MiCS 5135	1	Carbon Monoxide, Hydrocarbons, VOCs

**Table 2.** Time constants calculated for the rise and decay phase of each sensors.

**Sensor**	**T.C. Response**	**T.C. Decay**	**Fisher Index**
TGS 2600(1)	5:22 *s*	19:31*s*	32:85
TGS 2602	4:84 *s*	36:25 *s*	38:10
TGS 2611	3:52 *s*	7:36 *s*	10:99
TGS 2620	3:29 *s*	15:58 *s*	16:36
TGS 2600(2)	5:19 *s*	19:74 *s*	30:09
MiCS 2610	4:96 *s*	14:92 *s*	13:22
MiCS 2710	17:18 *s*	23:69 *s*	46:83
MiCS 5521(1)	2:43 *s*	5:54 *s*	2:65
MiCS 5121	5:97 *s*	10:72 *s*	19:59
MiCS 5135	5:37 *s*	14:65 *s*	17:23
MiCS 5521(2)	3:13 *s*	5:20 *s*	4:57

**Table 3.** True alarm ratio (TAR) and mean delay of detection (MDD) obtained when the false alarm ratio (FAR) is set to 0.1 for the single sensors.

**Model**	**TH**	**All**		**Change Concentration**		**Change Compound**		**Change Mixture**	
		TAR	MDD	TAR	MDD	TAR	MDD	TAR	MDD
TGS 2600 (1)	82	0.61	22.77	0.79	25.41	0.72	24.38	0.23	17.13
TGS 2602	79	0.41	24.94	0.52	26.81	0.40	34.05	0.22	15.98
TGS 2611	93	0.71	26.71	0.82	20.41	0.80	21.05	0.44	41.28
TGS 2620	93	0.61	25.94	0.76	32.30	0.69	23.24	0.29	16.52
TGS 2600 (2)	85	0.60	23.87	0.79	28.32	0.67	23.64	0.24	16.2
MiCS 2610	90	0.81	22.06	0.79	24.42	0.97	11.01	0.74	24.84
MiCS 2710	102	0.83	31.35	0.84	33.24	0.94	23.49	0.76	32.97
MiCS 5521(1)	90	0.59	24.93	0.76	20.94	0.66	22.98	0.25	33.14
MiCS 5121	91	0.76	22.46	0.83	19.20	0.82	18.96	0.62	30.36
MiCS 5135	77	0.84	20.09	0.83	20.33	0.98	13.05	0.78	24.08
MiCS 5521(2)	85	0.69	23.61	0.81	18.31	0.77	24.45	0.44	32.37

**Table 4.** Selected sensor subsets for the various parameters configuration.

*α*	*β*	**Specification**	**Selected Sensors**
0.9	0.5	fast, discriminative	MiCS 2710-MiCS 5521(2)
0.66	0.5	fast, discriminative, uncorrelated	MiCS 2710-MiCS 5521(2)-TGS 2602
0.5	1	fast, uncorrelated	MiCS 5521(2)-TGS 2602-MiCS 2610
0.5	0	discriminative, uncorrelated	MiCS 2710-TGS 2602
0.1	-	uncorrelated	MiCS 2610-TGS 2611-TGS 2602

**Table 5.** True alarm ratio (TAR) and mean delay of detection (MDD) obtained when the false alarm ratio (FAR) is set to 0.1 for the selected subsets of the sensor array.

**Model**	*h*	**All**		**Change Concentration**		**Change Compound**		**Change Mixture**	
		TAR	MDD	TAR	MDD	TAR	MDD	TAR	MDD
MiCS 2710-MiCS 5521(2)	151	0.83	24.79	0.85	20.43	0.93	19.95	0.75	35.45
MiCS 2710-TGS 2602	156	0.80	29.22	0.81	29.52	0.93	21.47	0.70	33.54
MiCS 2710-TGS 2602-MiCS 5521(2)	200	0.83	25.04	0.85	22.44	0.94	18.32	0.72	33.80
MiCS 5521(2)-TGS 2602-MiCS 2610	190	0.80	21.48	0.83	19.43	0.93	14.17	0.65	29.62
MiCS 2610-TGS 2611-TGS 2602	201	0.80	22.63	0.84	20.67	0.95	14.17	0.66	31.35

## References

[1] Baetz W., Kroll A., Bonow G. Mobile robots with active IR-optical sensing for remote gas detection and source localization.

[2] Hernandez Bennets V., Lilienthal A.J., Neumann P., Trincavelli M. (2012). Mobile robots for localizing gas emission sources on landfill sites: Is bio-inspiration the way to go?. Front. Neuroeng..

[3] Rachkov M., Marques L., Almeida A. (2005). Multisensor demining robot. Auton. Robot..

[4] Lilienthal A.J., Duckett T., Werner F., Ishida H. Indicators of gas source proximity using metal oxide sensors in a turbulent environment.

[5] Hayes A., Martinoli A., Goodman R. (2002). Distributed odor source localization. IEEE Sens. J..

[6] Ishida H., Wada Y., Matsukura H. (2012). Chemical sensing in robotic applications: A review. IEEE Sens. J..

[7] Trincavelli M., Coradeschi S., Loutfi A. Classification of odours for mobile robots using an ensemble of linear classifiers.

[8] Trincavelli M., Loutfi A. Feature selection for gas identification with a mobile robot.

[9] Roberts P., Webster D. (2002). Turbulent Diffusion. Environmental Fluid Mechanics Theories and Application.

[10] Trincavelli M. (2011). Gas discrimination for mobile robots. Künstliche Intelligenz.

[11] Basseville M., Nikiforov I.V. (1993). Detection of Abrupt Changes: Theory and Application.

[12] Aroian L.A., Levene H. (1950). The Effectiveness of Quality Control Charts. J. Amer. Stat. Assoc..

[13] Gustafsson F. (1996). The marginalized likelihood ratio test for detecting abrupt changes. IEEE Trans. Automatic Control.

[14] Adams R.P., MacKay D.J. (2007). Bayesian Online Changepoint Detection.

[15] Desobry F., Davy M., Doncarli C. (2005). An online kernel change detection algorithm. IEEE Trans. Signal Process..

[16] Wang X.R., Lizier J.T., Obst O., Prokopenko M., Wang P. (2008). Spatiotemporal anomaly detection in gas monitoring sensor networks. Lecture Notes Comput. Sci..

[17] Nakamoto T., Yoshikawa K. (2006). Movie with scents generated by olfactory display using solenoid valves. IEICE Trans. Fundam. Electron. Commun. Comput. Sci..

[18] Figaro Engineering Inc http://www.figarosensor.com/.

[19] e2v Technologies, Inc http://http://www.e2v.com/.

[20] Holt C.C. (2004). Forecasting seasonals and trends by exponentially weighted moving averages. Int. J. Forecast..

[21] Gutierrez-Osuna R. (2002). Pattern analysis for machine olfaction: A review. IEEE Sens. J..

[22] Phaisangittisagul E., Nagle H.T., Areekul V. (2010). Intelligent method for sensor subset selection for machine olfaction. Sens. Actuators B Chem.

[23] Guyon I., Elisseeff A. (2003). An introduction to variable and feature selection. J. Mach. Learn. Res.

[24] Rodriguez-Lujan I., Huerta R., Elkan C., Santa Cruz C. (2010). Quadratic programming feature selection. J. Mach. Learn. Res.

